# Efficacy of Bioactive Cyclic Peptides in Rheumatoid Arthritis: Translation from In Vitro to In Vivo Models

**DOI:** 10.3390/molecules22101613

**Published:** 2017-09-25

**Authors:** Roger New, Michal Bogus, Gurpal S. Bansal, Malgorzata Dryjska, Katarzyna Zajkowska, Michael Burnet

**Affiliations:** 1 Proxima Concepts Limited, c/o London Bioscience Innovation Centre, 2 Royal College Street, London NW1 0NH, UK; michalbogus@proximaconcepts.com (M.B.); gurpal.bansal@adaptimmune.com (G.S.B.); gosiadryjska@wp.pl (M.D.); kz@cantab.net (K.Z.); 2 Adaptimmune Ltd., 60 Jubilee Ave., Milton Park, Abingdon OX14 4RX, UK; 3 Cambridge Blue Ventures Ltd., 21 Arlington St, London SW1A 1RN, UK; 4 Synovo GmbH, Paul Ehrlich Str. 15, 72076 Tübingen, Germany; michael.burnet@synovo.com

**Keywords:** cyclic peptide, TNF, interleukin 6, rheumatoid arthritis, lipoamino acid

## Abstract

Using a novel drug discovery technology reported in previous issues of this journal cyclic peptides have been created which are able to down-regulate secretion of inflammatory cytokines, in vitro, by stimulated cells of the macrophage cell line J774. The cytokines in question, TNF-alpha and IL-6, are strongly implicated in etiology of diseases such as rheumatoid arthritis. Studies are reported here using the CAIA animal model for rheumatoid arthritis, which show that the peptides identified are indeed able to impact on inflammation of joints, induced in vivo. The results suggest that these peptides are effective at a dose which could be viable in man, and at which no adverse side effects are evident in the short term.

## 1. Introduction

Drug molecules can have many striking effects when tested in vitro in cell culture models, but, for many reasons, there is no guarantee that these effects will translate into comparable therapeutic activity in an in vivo experimental model. Confounding factors include the unusual behaviour of cultured animal cell lines, different time courses of activity between a cell monolayer and a whole organism, drug interactions with blood components absent from culture media, restricted access to relevant cells in vivo, and unwanted side effects in normal cells in vivo. In the case of the peptides studied here, proof-of-principle was demonstrated in both a murine cell line, and human primary isolates [1,2]. However, culture conditions reflected the in vivo situation only approximately, peptide concentrations being maintained at a high concentration for eighteen hours, the stimulation of cells taking place two hours after introduction of the peptide, and the culture medium containing a relatively low concentration of serum components (~10% foetal bovine serum). In addition, culture media contain none of the agents which are commonly responsible for degradation in vivo of drugs, particularly peptides.

Thus, a very important part of the development process for any potential therapeutic agent is a clear transfer from in vitro to in vivo models, and in the case of the peptides described here, this transition was conducted in three stages. The first stage was to try and create a situation in a rodent as close as possible to the ‘test-tube’ culture conditions already employed. In this first experiment, the same stimulus was employed (lipopolysaccharide—LPS), and the same outcome monitored (TNF secretion, and inhibition thereof). As part of the discovery process, micelles were first created with prototype epitopes on their surfaces, before progressing to micelle-free cyclic peptides. In the LPS-stimulated rat model, both types of agent (micelle and cyclic peptide) were tested, in order to compare with the positive results which both agents had demonstrated in vitro.

The second and third stages involved the use of a standard model for rheumatoid arthritis in mice—the Collagen Antibody-Induced Arthritis (CAIA) model [3,4,5]. This was first performed with the peptides given after the first stimulus, and then repeated with the stimulus given shortly before.

Rheumatoid arthritis (RA) is a disease which appears to have existed from ancient times, as bone erosions reminiscent of RA are evident in skeletons from Egyptian archaeological sites dating back at least to 1000 BC [6]. It was first described in modern times by Landré-Beauvais in 1800 [7], and the pathology described in detail by Garrod [8] in 1859. From the late 1950s onwards, with the advent of sophisticated immune-diagnostic techniques, an immune component of the disease was recognised, with patients commonly being shown to have high levels of auto-antibodies in their tissues and blood, initially antinuclear antibodies [9], and later anti-collagen antibodies [10]. The ability to induce symptoms of rheumatoid arthritis in experimental animals after administration of collagen type II in adjuvant [11], and later, antibodies to collagen type II [12], lends support to the idea that these antibodies play a causative role in the pathology of the disease, and such models form the basis of the study of rheumatoid arthritis and its treatment to this day. The reason for the appearance of the antibodies in the first place, however, is still not clear, and may be due to a combination of factors relating to genetic predisposition, as well as pre-exposure to other causative agents, including certain infectious diseases.

Antibodies complexed with auto-antibodies give rise to inflammatory reactions, which are often self-regulating, but which, in the arthritic joint, appear to be perpetuated by a mutual stimulatory interaction between activated macrophages and T cells, probably of the Th17 phenotype, generated in the presence of the specific cocktail of cytokines present in inflamed synovial joints [13,14,15]. One of the major cytokines whose level is elevated in RA is tumour necrosis factor (TNF), and the key role that TNF plays in this scenario is highlighted by the fact that reduction of TNF levels alone, in the bloodstream and other body fluids, using anti-TNF monoclonal antibodies, is enough to reduce dramatically the symptoms of rheumatoid arthritis in the clinic [16]. TNF blockade, using anti-TNF antibody-based molecules such as Humira^®^ (adalimumab), Remicade^®^ (infliximab) or Enbrel^®^ (eternacept), is now a widely used approach to treatment for rheumatoid arthritis [17,18,19], and while it is extremely effective in many patients, there are a significant number of non-responders, and symptoms often return after treatment is discontinued. Searches for additional approaches to treatment of rheumatoid arthritis are thus continuing.

In the approach taken here, rather than try to remove TNF from the tissues after it has been secreted, the aim is to act on cells in order to prevent its being secreted in the first place. Evidence to be presented separately suggests that one of the peptides studied here, Lex 2.6, inhibits secretion from macrophages alone, and has no effect on secretion by lymphocytes. Retention of the ability of lymphocytes to secrete TNF could be important in avoiding some of the side-effects seen with standard TNF blockage. Two conditions known to be associated with anti-TNF antibody treatment of rheumatoid arthritis, namely increased infection with *M. tuberculosis* and occurrence of Merkel cell carcinomas, have both been identified as being kept at bay by TNF-secreting lymphocytes [20,21], respectively.

## 2. Results

**Figure 1:**
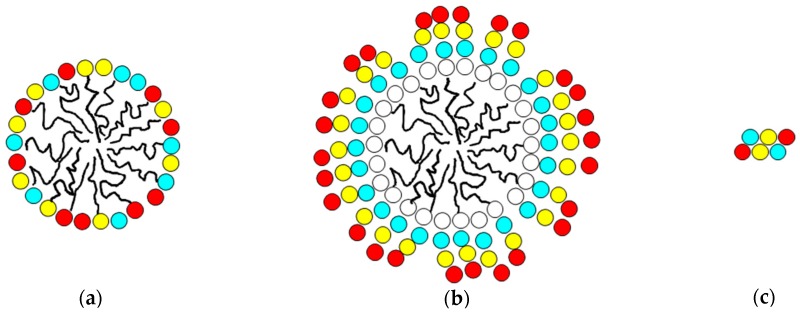
Representations of the different ways in which amino-acid combinations are presented. (**a**) as individual headgroups on the surface of a micelle; (**b**) as linear peptides on the micelle surface and (**c**) as self-contained cyclic peptides independent of micelles.

The process of implementation of the Mozaic™ discovery technology involves screening of a library of micelles, each presenting a different combination of single amino acids on their surface [1]. In this study, the micelle which was most effective in down-regulating TNF secretion was one which comprised S, F and R amino acids. The technology then seeks to develop a small oligo-peptide containing the same amino acids, but which can present them as a rigid ligand-binding epitope to interact with cell receptors without need for presentation on a micelle surface. The end result (an internally constrained cyclic peptide) is described elsewhere [2], and examples are shown in Figure 6 below. However, in certain circumstances, a “halfway house” is introduced into the process, in which peptides are constructed, but these peptides are still presented on the surface of micelles. This reduces the risk of failure in the later stages, and gives confidence that the strategy adopted is a reasonable one. The three stages are illustrated in Figure 1.

In order to produce micelles presenting linear peptides (conforming to the structure shown in Figure 1b), amphiphiles were synthesised in which linear peptides are attached at the C-terminal end to lipid anchors via a three-amino acid spacer group (in this case hydroxyproline-serine-glycine). The lipid anchor, in common with the more traditional micelles used to present single amino-acid headgroups (as in Figure 1a) were formed of two amino acids with extended C10 lipid straight chain side groups. Even with only three amino acids, there are six possible ways of putting them together in a linear sequence, and all of these were synthesised and compared. The different amphiphiles are shown below, code-named A–F. Glycolic acid (‘glyc’) is employed to block the N-terminal amino group with a neutral hydrophilic replacement. C12 refers to an amino acid with a ten-carbon straight hydrocarbon chain as the side-chain residue. The peptide link between these two amino acids is *N*-methylated (indicated as ‘Nme’ below).

A: glyc-F-R-S-hyp-ser-gly-C12-Nme-C12B: glyc-S-F-R-hyp-ser-gly-C12-Nme-C12C: glyc-R-S-F-hyp-ser-gly-C12-Nme-C12D: glyc-F-S-R-hyp-ser-gly-C12-Nme-C12E: glyc-R-F-S-hyp-ser-gly-C12-Nme-C12F: glyc-S-R-F-hyp-ser-gly-C12-Nme-C12

**Figure 2:**
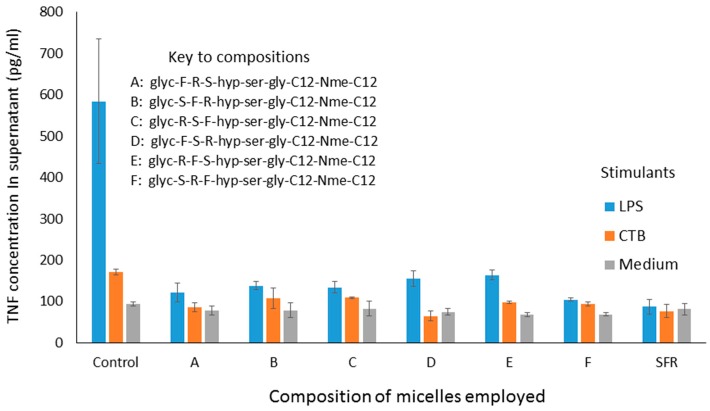
TNF levels in supernatants of cultured J774A.1 murine macrophage cells after exposure to Mozaic FSR variants mixed micelles (60 μg/mL) administered four hours prior to stimulation by Cholera Toxin B (CTB) fragment (10 μg/mL) or lipopolysaccharide (LPS, 60 ng/mL). TNF levels were measured 18 h after stimulation of the macrophages. The group ‘SFR’ refers to the micelle with single amino acids as headgroups.

The ability of micelles comprising these amphiphiles to inhibit secretion by J774 cells of TNF-alpha stimulated either by LPS or CTB is shown in Figure 2. It should be noted that the results described here in Figure 2 and Figure 4 show the use of different stimuli (CTB and LPS). These were included in the same graph because the data were obtained from the same experiment, so that the control is the same for both. We felt that it was an important observation that, although these stimuli are indeed distinct, the peptides affect the outcome for both in the same way, indicating an underlying mechanism which relates to a point where these two stimulatory pathways merge. Compared with LPS, CTB is much less active at stimulating TNF secretion.

Interestingly, all of these micelles clearly inhibit TNF secretion, and there appears to be no marked difference between the efficacy of any of them. This is somewhat surprising, until one realises that, because of the fact that these peptides are presented on the surface of micelles, it is probable that receptors being targeted bind not to just one three-amino-acid sequence, but have a choice of binding to different parts of closely adjacent sequences, so that the receptor interacts with the three amino acids in the optimal configuration, regardless of the nature of the individual sequence. A second experiment, in which different sequences were combined in the same micelle also showed inhibition, although no greater than with the single amino acid sequences alone (data not shown). In a third experiment, one of the sequences (S-F-R) was selected for further study, and amphiphiles were synthesised, in which the anchors were different, as shown below. In all cases, SFR here indicates the linear sequence.

Moz.2-3:glyc-SFR-hyp-ser-gly-C12-Nme-C12Moz.2-3 N:glyc-SFR-hyp-ser-gly-C6 (single)Moz.3-3:glyc-SFR-hyp-ser-gly-C12 (single)Moz.5-1:glyc-SFR-hyp-ser-gly-C14 (single)

**Figure 3:**
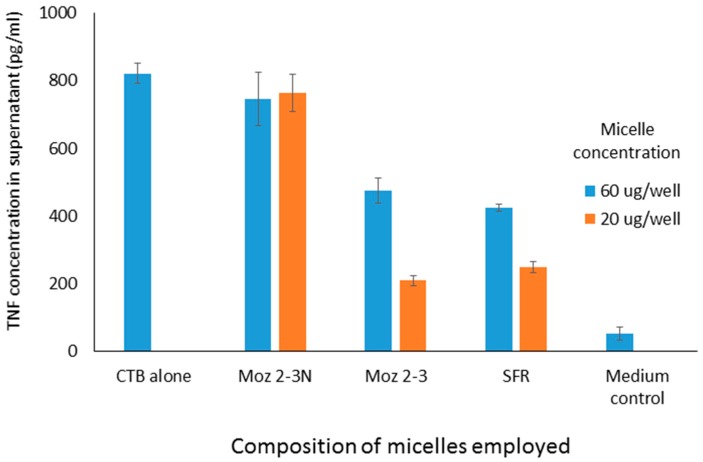
TNF levels in supernatants of cultured J774A.1 murine macrophage cells after exposure to amphiphilic lipopeptides containing different lipid chains, but all possessing the same S-F-R linear peptide headgroup. Test substances were placed in the wells four hours prior to stimulation by CTB (10 μg/mL). TNF levels were measured 18 h after stimulation of the macrophages. Moz 2-3 contains two C12 lipidic amino acids as anchors. Moz 2-3N contains just one single nor-leucine.

**Figure 4:**
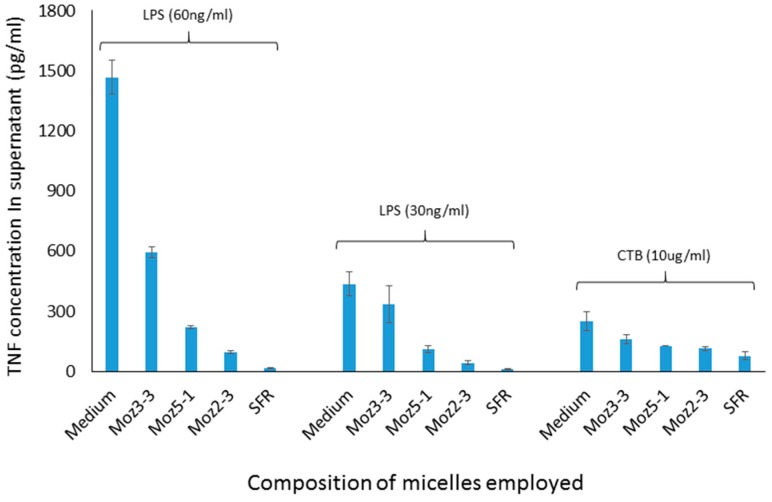
TNF levels in supernatants of cultured J774A.1 murine macrophage cells after exposure to amphiphilic lipopeptides containing different lipid chains, but all possessing the same S-F-R linear peptide headgroup. Test substances were placed in the wells four hours prior to stimulation by CTB or LPS. TNF levels were measured 18 h after stimulation of the macrophages. Moz 2-3 contains two C12 lipidic amino acids as anchors. Moz 3-3 contains a single C12 amino acid, while Moz 5-1 contains a single C14 amino acid.

It is worthy of note that the anchor composed just of a single C6 straight chain amino acid (nor-leucine) is one which is expected not to form micelles at all upon dispersion in aqueous phase. As can be seen in Figure 3, using a cell culture model involving stimulation of J774 cells by CTB, dose dependent inhibition of TNF secretion was observed for both micelles containing individual amino acids, and those containing the linear sequence, while a dispersion of the nor-leucine conjugated SFR sequence had essentially no activity (i.e., no inhibition). With the same headgroup, in order to determine more precisely where the cut-off lies between an efficacious micelle and a non-effective lipopeptide aggregate, additional amphiphiles were constructed comprising the S-F-R sequence attached by a hyp-ser-gly spacer to a single lipoaminoacid containing either twelve carbons in total, or fourteen. The efficacy of dispersions of these peptides was compared in culture with cells stimulated either with CTB or LPS. In both cases (see Figure 4) a single C14 chain was sufficient to manifest the inhibitory effect of the S-F-R sequence, but a C12 chain was less active. It is worth noting that, in separate experiments (not shown here) micelles were administered not only before but also after exposure of the cells to the inflammatory stimulants. In the latter case, inhibitory effects on TNF secretion were also observed, although not as marked as when introduced prior to the stimulants. Since the purpose of the experiments described here was to distinguish between the magnitude of effects of the different micelle and peptide sequences, it was felt that exposure of cells to test reagents prior to stimulation was an acceptable model to use.

Micelles composed of the same S-F-R sequence as employed above were tested in an in vivo model, paralleling closely the in vitro model employed above. As described in detail in the materials and methods section, six groups of six rats each received different test samples i.p., followed shortly afterwards by LPS. Concentrations of TNF in the bloodstream were measured by ELISA at different times afterwards. The samples tested are shown below.

A—Saline vehicle followed by saline i.p.
B—Micelles comprising individual S, F and R amino acids followed by saline i.p.
C—Micelles comprising linear S-F-R peptide followed by saline i.p.
D—Saline vehicle followed by LPS i.p.
E—Micelles comprising individual S, F and R amino acids followed by LPS i.p.
F—Micelles comprising linear S-F-R peptide followed by LPS i.p.

**Figure 5:**
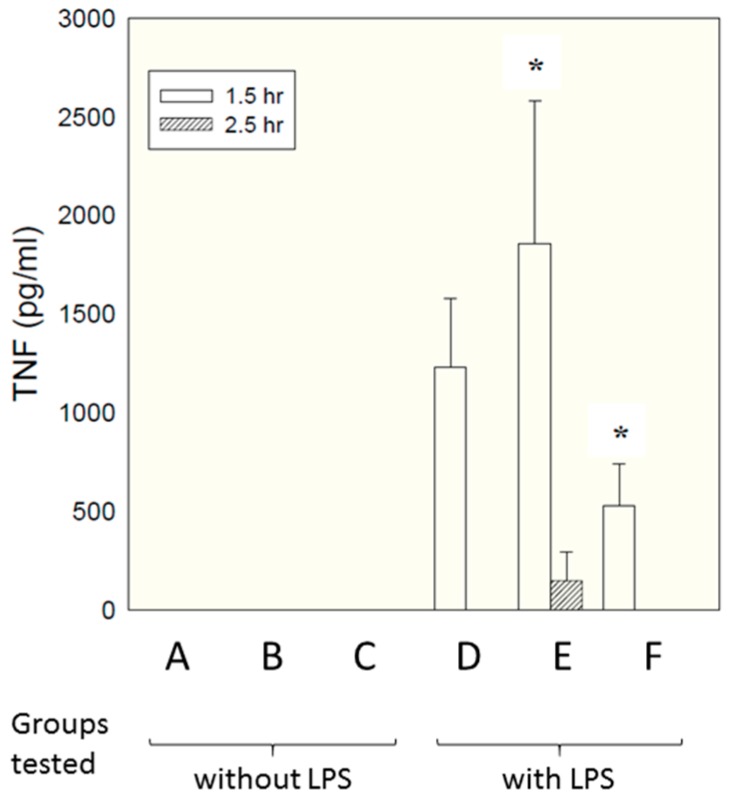
Effect of micelles on plasma TNF levels. Plasma TNF levels were measured by ELISA after 1.5 h and 2.5 h in all rats (6 per group) and expressed as pg/mL. Results are shown as mean ± SD. * indicates *p* < 0.05 (compared to group D) at 1.5 h using ANOVA followed by Student-Newman-Keuls statistical test. A—Saline vehicle followed by saline i.p.; B—Micelles comprising individual S, F and R amino acids followed by saline i.p.; C—Micelles comprising linear S-F-R peptide followed by saline i.p.; D—Saline vehicle followed by LPS i.p.; E—Micelles comprising individual S, F and R amino acids followed by LPS i.p.; F—Micelles comprising linear S-F-R peptide followed by LPS i.p.

Figure 5 shows TNF levels measured at time 90 min after administration of LPS.

Interestingly, a significant inhibitory effect was seen for the micelles presenting the epitopes as linear peptides on their surface (group F, compared with group D—LPS alone), while micelles presenting just single amino acids (group E) enhanced TNF secretion. This is attributable to the fact that, in contrast to in vitro culture conditions, in vivo there are many factors encouraging dismemberment of micelles—interactions with chylomicrons, lipoproteins, fatty deposits, albumin etc. Micelles with single amino acid headgroups appear to have insufficient cohesiveness to maintain their integrity in the face of such influences—possibly simply because inter-chain hydrogen bonding is less extensive than for the linear sequence, which contains a larger number of peptide linkages capable of hydrogen bonding. Group F also showed a significant reduction in weight loss compared with LPS alone (5.8% compared with 8.4%, respectively. Data not shown). There was no effect of either compound on peritoneal leukocyte numbers; although the linear compound decreased LPS-induced polymorpho-nuclear-leucocyte extravasation score in the liver and lung by 30–50%, this failed to reach statistical significance (data not shown).

**Figure 6:**
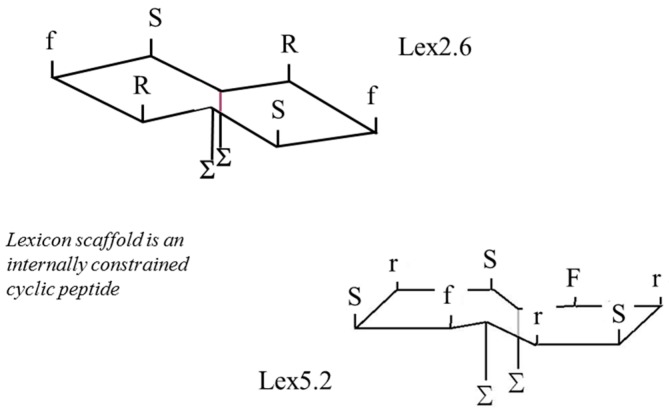
Schematic of structure of Lexicon peptides employed.

These results demonstrate clearly that, although micelles are good at presenting epitopes to cells in culture, there may be significant issues related to their use in vivo. For this reason, peptides were created in which the same amino acid side-chains were presented on a planar surface similar to that presumed to pertain on the exterior of the micelle. The development process is described elsewhere [1,2], and the resultant peptides (described as ‘Lexicon’ peptides) are shown in Figure 6, where two lipid chains (represented by ‘Σ’ on the opposite side from the S, F and R side-chain residues) are enclosed inside the cavity of a hydroxypropyl β-cyclodextran ring. Upper and lower case letters indicate L and D chiral forms, respectively.

**Figure 7:**
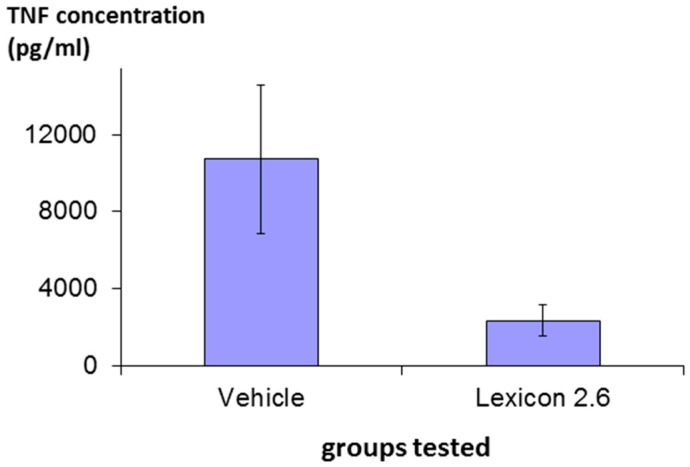
Effect of Lex 2.6 on plasma TNF levels. Plasma TNF levels were measured by ELISA after 1.5 h in all rats (6 per group) and expressed as pg/mL. Results are shown as mean ± SD.

The efficacy of structure Lex 2.6 was confirmed in vivo following a protocol essentially identical to that described above in rats stimulated with LPS, and the result is shown in Figure 7, where it can be seen that the molecule has marked inhibitory activity.

**Figure 8:**
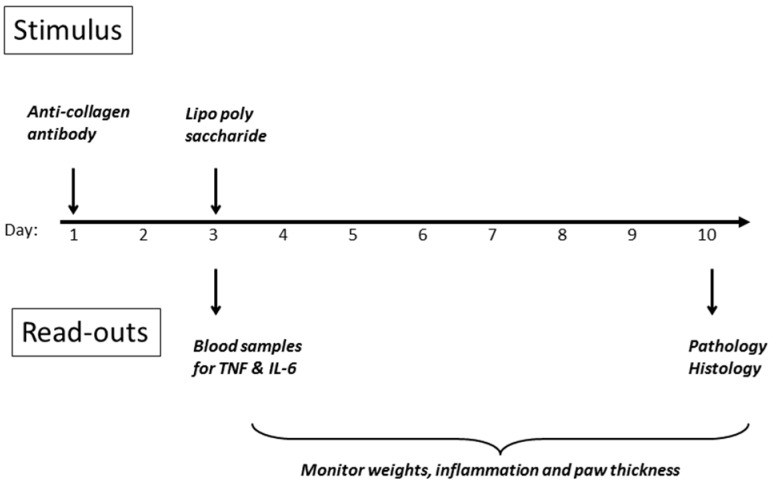
DBA mouse model for inflammatory disease: Collagen Antibody-Induced Arthritis (CAIA).

In light of the ability of cyclic peptides to alter the behaviour of macrophages, down-regulating TNF secretion both in vitro and in vivo, the investigation was extended to examine whether such peptides could have an impact on inflammatory diseases such as rheumatoid arthritis, where activation of macrophages, and secretion of TNF, is an important component. The CAIA model [22,23] was chosen, as its use of lipopolysaccharide as one of the stimulants mirrors the models already employed. The other inducer of inflammation is a cocktail of anti-collagen antibodies, which are agents thought to contribute to disease in the human condition. A scheme of the experimental design is shown in Figure 8, and detailed methodology is provided in the Materials and Methods section of this article. The initial stimulus is a cocktail of anti-collagen antibodies administered s.c. in the dorsal region on day 1, followed by a non-specific inflammatory stimulus two days later, whereupon inflammation in all four paws develops rapidly, and is maintained for up to ten days before resolving spontaneously. DBA/2 mice were employed (10 per group) since they are genetically predisposed to development of the symptoms of rheumatoid arthritis, when challenged in the appropriate manner.

Treatment with peptide commenced on day 0, one day prior to the initial stimulus on day 1. In the case of the anti-TNF antibody (Humira), the whole dose was given as a bolus on day 1. For the peptide, the dose was divided over ten days, so that the final dose of peptide was not received until close to the end of the experiment. An internal control using the well-known anti-inflammatory agent dexamethasone was also run, which confirmed the consistency of the model.

In the first part of the study, a range of doses of peptide was tested (see Table 1 below), after which it was apparent that the dose range employed was not broad enough. Further doses (marked with an asterisk in the table below—one higher and one lower than the original range spanned) were tested in animals from the same cohort, approximately two weeks later.

A range of different parameters was measured, as follows: (i) clinical score—a subjective assessment of the number of joints in each paw affected; (ii) paw thickness—measurement of oedema in the paws, reflecting the severity of inflammation; (iii) TNF levels—rise in the bloodstream immediately after LPS administration; (iv) weight change—loss of weight resulting from inflammation.

**Figure 9:**
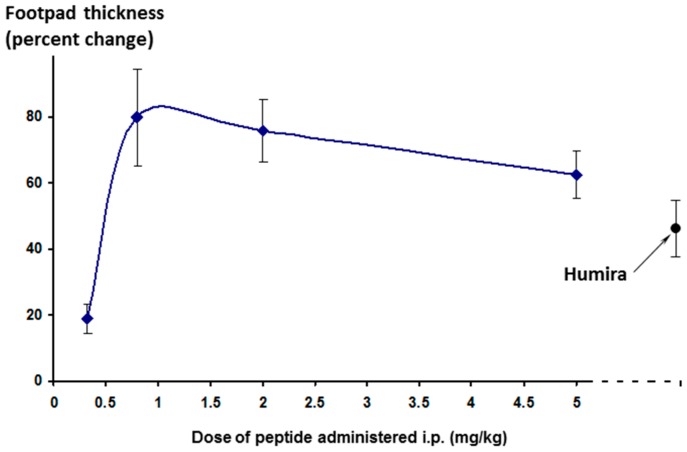
Effect of different doses of Lex 5.2 cyclic peptide on inflammation in the CAIA model, as measured by footpad swelling. Percent change is calculated relative to the difference between naïve animals (no inflammatory stimulus) and treated animals (stimulated but receiving just the vehicle control). 100% is maximal disease, 0% is no disease manifestation. The lower the value, the greater the reduction of inflammation.

The effect on footpad thickness (the primary measure of inflammation) is shown in Figure 9 for a range of doses tested. Interestingly, the greater efficacy was seen for the lowest dose tested (0.32 mg/kg/day)—a phenomenon known as hormesis [24], which is often observed with peptides. One additional higher dose was also tested (15 mg/kg/day, discussed later).

**Figure 10:**
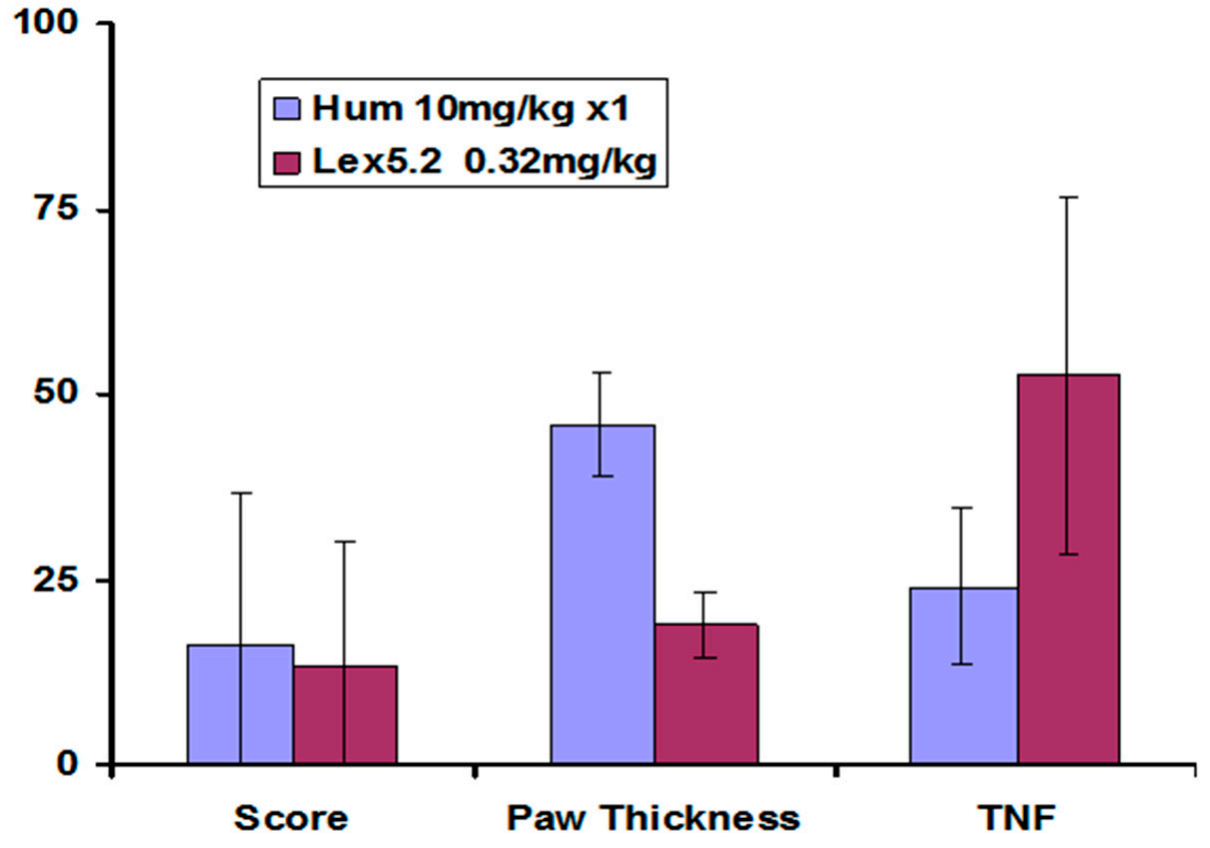
Effect of Lex 5.2 cyclic peptide, compared with Humira anti-TNF antibody, on key parameters of inflammation in the CAIA model, at the most efficacious dose—0.32 mg/kg/day (administered on 10 successive days). Values are percent change relative to naïve animals: Vehicle control is 100% in each case. Error bars are standard deviations. Clinical score and paw thickness were measured at day 10. The TNF levels were measured 90 min after LPS injection. Maximal disease is 100%. The lower the value, the greater the reduction in inflammation.

Other parameters measured also showed greatest change at the lowest dose, and these are compared at that dose in Figure 10.

**Figure 11:**
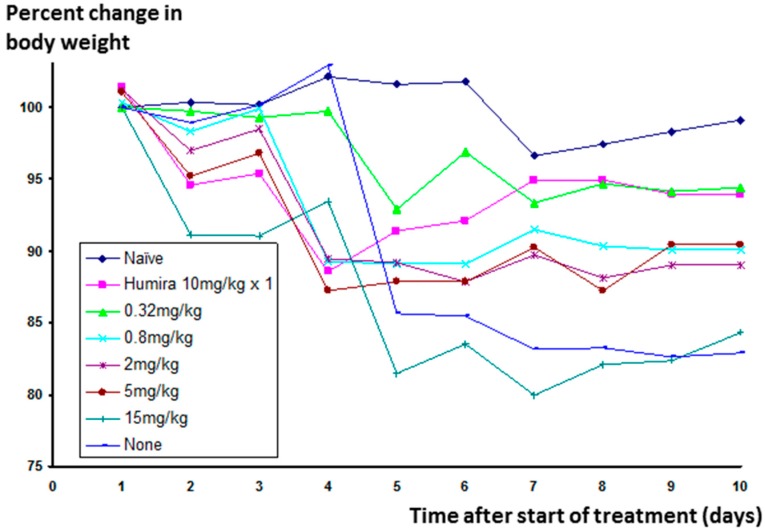
Effect of Lex 5.2 cyclic peptide at different dose levels on weight change in mice in the CAIA model, over a ten-day period.

Significant weight loss occurs in animals in which inflammation has been stimulated, but not treated. The change in weight for animals receiving Humira^®^ was much reduced, and Lex 5.2 at the 0.32 mg/kg dose level gave an identical result (See Figure 11 below).

**Figure 12:**
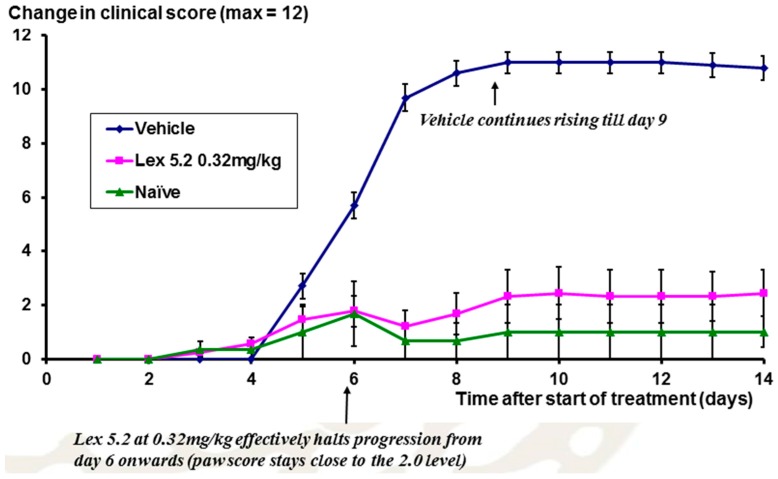
Time course of inflammation in the CAIA model, over a ten-day period, expressed in terms of clinical score. Error bars are standard errors.

The time course of the disease is shown in Figure 12, looking at the change of clinical score over time. From day 5 onwards, scores rise as a result of the LPS stimulation on day 3, but at day 6 for Lex 5.2 (0.32 mg/kg), the rise in clinical score is halted, and is not significantly different from naïve animals.

**Figure 13:**
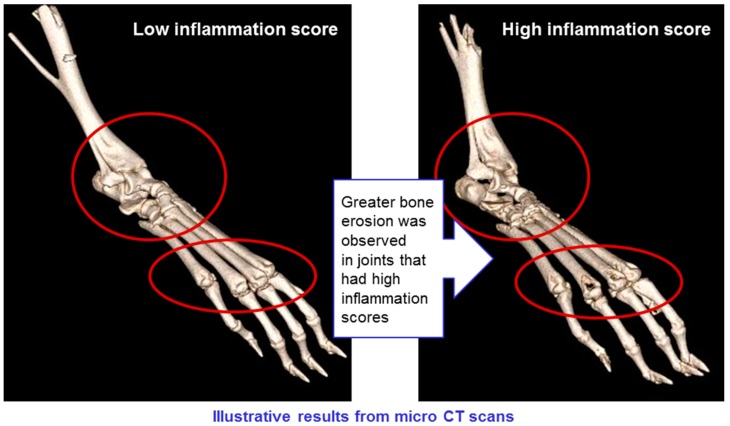
Model validation: Bone erosion correlates with inflammation scores.

CT scans of mice in all groups were conducted, showing erosion of bone in animals with high levels of inflammation, but not in those where inflammation was low (see Figure 13). This confirms that the CAIA experimental system is a valid model for rheumatoid arthritis, and that the treatments have a positive impact on all manifestations of the disease.

One higher dose was tested (15 mg/kg/day) in the second part of this study in which, although little efficacy was seen, signs of mild toxicity were observed. No fatalities occurred, even at this high dose, which was approximately 50 times higher than the most efficacious dose level, and no toxicity of any sort was seen with any of the other doses. Observations were made of pale liver and adhesion of intestine to the wall of the abdominal cavity, which could have been associated with the intraperitoneal route of administration, and not the API. Body weights of the group treated with the lowest dose were stable at the end of the experiment, and similar to naïve animals.

**Figure 14:**
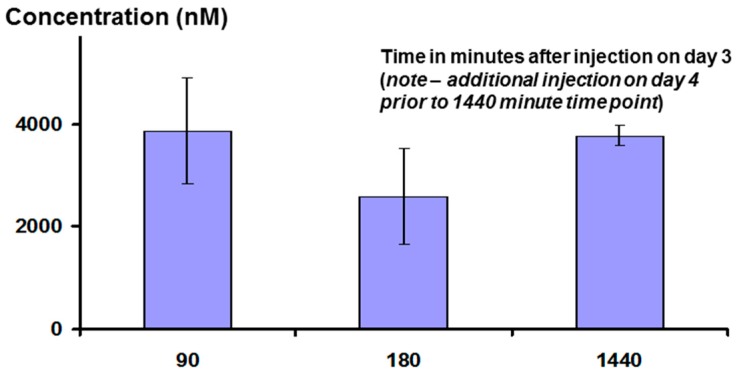
Concentration of Lex 5.2 peptide in the bloodstream at different times (in minutes) after intraperitoneal injection (15 mg/kg).

Interestingly, measurement of plasma peptide levels after i.p. administration, using LCMS as the assay method, showed that the peptide stays in the bloodstream for a significant length of time, with a half-life of clearance probably between three and six hours (see Figure 14 below). This may be the result of association between the peptide and albumin, or lipoproteins.

A follow-up study was conducted to obtain additional data not generated in the first study. The study used the CAIA model again, and the following aspects were investigated:

1. Efficacy of additional dose levels, lower than those already tested.
2. Comparison with an anti-TNF antibody-based molecule other than Humira^®^, namely Enbrel^®^ (a fusion protein of the TNF receptor with antibody Fc portion).
3. Behaviour in this model of another member of the Lexicon series—Lex 2.6.

The experimental plan of the follow-up study was identical to the first, however with a few small differences. The inflammatory response took longer to mature, and so the observation period was extended by several days. In addition, TNF levels after LPS administration were surprisingly low, with no differences observed between groups. These departures were attributed to the fact that the supplier of the antibody cocktail had changed its make-up, including antibodies against two additional epitopes. Finally, the cyclic peptides were administered on day 1, one hour after injection of the inflammatory stimulus, instead of one day before, as in the previous study. This was done in order to separate the effect of the treatments on inflammation from any effect on the initiation step.

A range of dose levels of Lex 5.2 was tested, from 0.025 mg/kg up to 0.6 mg/kg (see Table 2 below). In addition Lex 2.6 was tested at a dose level similar to that which demonstrated efficacy with Lex 5.2 in the previous study.

**Figure 15:**
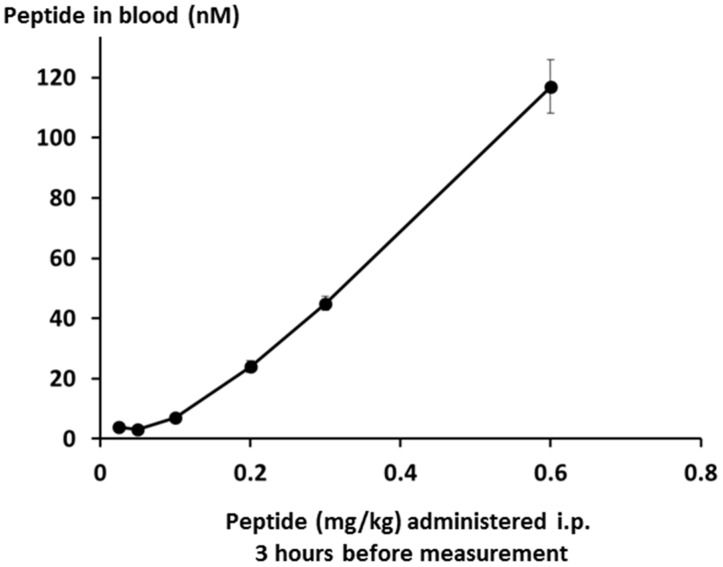
Lex 5.2 levels in the blood after intraperitoneal administration of different doses on day 3.

Peptide levels in the bloodstream were measured by LCMS, and were found to be linear over the whole dose range down to the limit of quantification as shown in Figure 15.

**Figure 16:**
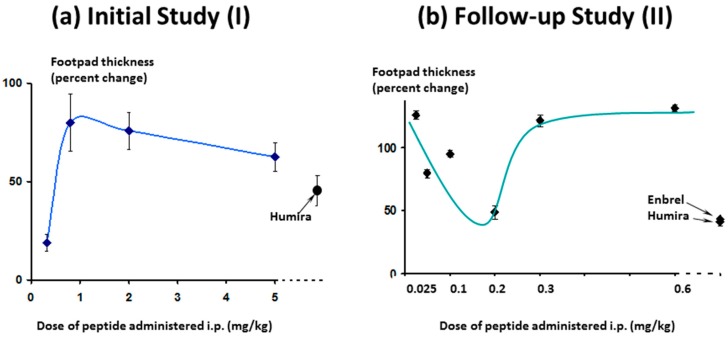
Effect of Lex 5.2 on inflammation: comparison of results from two studies. Values shown were measured just prior to the termination of the experiment.

As expected, in the follow-up study, efficacy increased as the dose approached 0.3 mg/kg/day and below, (Figure 16, right-hand panel) as was seen in the first study (Figure 16, left-hand panel). As the dose levels reduced further (right-hand panel) efficacy also reduced, as expected.

**Figure 17:**
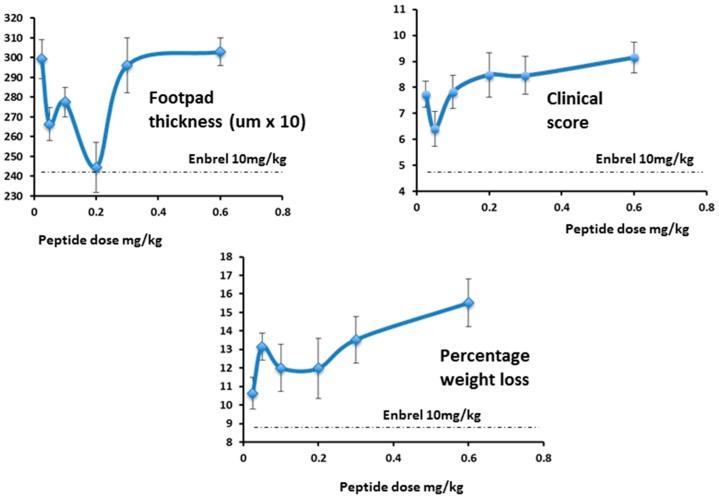
Dose-response curves for Lex 5.2.

While footpad thickness is considered to be the indicator most relevant to measurement of severity of an on-going inflammatory response, other parameters shown here (clinical score and weight change) followed a similar pattern (Figure 17). As expected, however, the effect of Lex 5.2 on clinical score is less marked than in the previous study, since the clinical score may be considered indicative of the initiation phase, and the peptide was administered after the initial stimulus had been given. The fact that a marked effect on footpad swelling is seen even when clinical score is less affected shows that the reduction of inflammation observed is not an artifact due to inhibition of the initiation phase.

**Figure 18:**
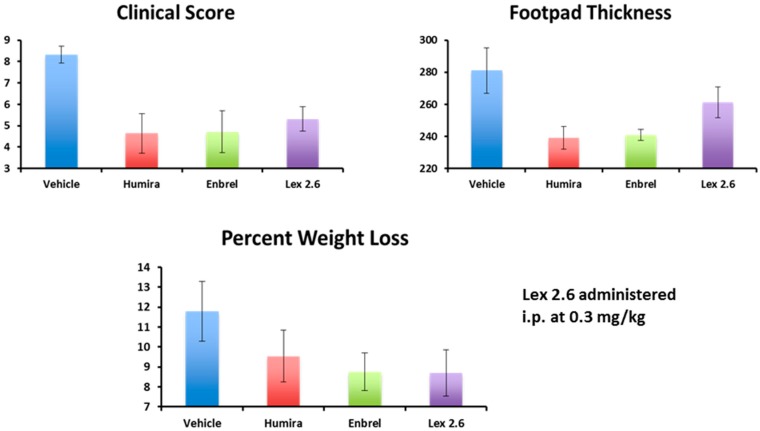
Comparison of Lex 2.6 with monoclonal antibody controls.

Interestingly, efficacy of Lex 2.6 at the dose level chosen was equivalent or better that Lex 5.2 for these parameters, and gave values close to both Enbrel^®^ and Humira^®^ (Figure 18). Although the dose-response curve suggests a rather narrow concentration window to achieve optimum efficacy, this is likely to be an artefact of the i.p. dosing regime, where small repeated doses tend to favour removal of peptide from the circulating pool before reaching its target. A higher initial dose, to saturate spurious binding sites, and administration in a depot by i.m. or s.c. injection should also help. Alternatively, oral administration, in the right vehicle, may be able to circumvent these spurious interactions altogether.

## 3. Discussion

The development programme described in the previous papers cited [1,2] indicated that a combination of F, S and R on a planar ring, arranged in a clockwise triangle was the motif which elicited the responses observed. We felt it was important to establish whether this could be presented on different types of ring, or was critically dependent on one sole conformation. This could have implications in the future on manufacturability, stability etc. In the event, it turned out that both peptide variants had efficacy, and is a very significant observation.

The peptide has been designed in such a way that it displays maximum possible rigidity, first of all through being cyclised, then engineered so that opposite sides of the ring come close together as a result of association of the side-chains of lipoamino acids with extended side-chains. As a corollary to this, hydrogen bonds form across the ring, stabilising it further. Finally, the solubility of the peptide is enhanced by complexing it with β-hydroxypropyl cyclodextrin, which masks the lipid chains and minimises their contact with water. At the same time, the cyclodextrin contributes further to the rigidity, and helps the peptide chain to form a planar structure, where the amino acid side-chains are all located above the plane, capable of interacting with receptors, while the lipid chains and cyclodextrin molecule are located beneath the plane of the peptide chain. In this way, the peptide mimics the orientation of the sidechains on the planar surface of the micelle, the presentation system that was originally employed to identify which amino acid combinations were active in bringing about biological changes (in this case inhibition of TNF secretion) in in vitro models for disease (see References [1] and [2] for more information).

More detailed characterisation of the peptide/cyclodextrin complex would be useful; this is currently being undertaken, and will be reported in future publications. Knowledge of the peptide structure, and an understanding of how cyclodextrin interacts with other lipophilic molecules, suggests that most likely a 1:1 complex of the two agents is formed, with the two hydrocarbon side-chains of the peptide being enclosed within the internal cavity of the cyclodextrin ring. It is not clear whether separation of the peptide from the cyclodextrin ring occurs in vivo, but this could be a highly desirable occurrence, since the peptide may then associate with blood components. If such an association were to take place via the lipid chains on the underside of the ring, leaving the upper side exposed and able to interact with receptors as before, this would be advantageous, since it would lead to the prolongation of the lifetime of the active peptide in the bloodstream. The normal half-life for peptides of this size in the bloodstream is about twenty minutes, where the molecule is excreted through the kidney. The fact that a much longer half-life is observed suggests that such binding to blood components is indeed taking place. Likely candidates for the peptide to bind to would be albumin or lipoproteins. In the case of the latter, it is possible that more than one peptide molecule could attach to the surface of a single lipoprotein, so that multiple copies of the peptide may present themselves to receptors on the surface of target cells, thus enhancing the initiation of a signalling cascade via this multivalent interaction, in the same way as has been proposed for the micelles.

Experiments in vitro [2] showed changes in TNF and IL-6, but none in IL-1 or IL-10. In the experiments described here, only changes in TNF levels were sought. It should be remembered that the appearance of cytokines in this CAIA model is often transient, and in some cases even TNF levels are not elevated (as in the second CAIA experiment conducted here), so there is no change to detect. The authors feel that, although initially it was the observation of TNF changes in vitro which led them to believe these peptides may influence the outcome of disease in an RA model, the underlying mechanism of action is more than just reduction of TNF in the body fluids, but probably a change in the activation status of the inflamed macrophages themselves. This is suggested by the fact that the same peptides inhibit secretion of TNF stimulated by both CTB and LPS—two very different stimulatory mechanisms, but which both transform quiescent macrophages into activated ones. Further work is underway to identify the receptor through which the peptides are acting, to look at which cell populations are being affected by the peptides, and whose behaviour is being changed, and finally to establish the effect that these peptides have on an inflammatory reaction which is already on-going. This will require a different, more chronic model, since the CAIA model in mouse is rapid onset, but also rapidly self-resolving.

## 4. Conclusions

An important conclusion is that, despite their small size, the internally-constrained cyclic octa- and deca-peptides designed according to the principles described here are capable of eliciting biological responses in vivo with significant therapeutic implications, and that this capability is in major part due to the high degree of rigidity built in to the peptides, and the planar topographical distribution of the side-chain residues. The data generated to date show that the two lead compounds demonstrating efficacy in the original in vitro studies both have significant impact on parameters indicative of efficacy in rheumatoid arthritis and other inflammatory diseases. Reductions in TNF levels are seen for the peptides at all dose levels, but these did not correlate with reduction in inflammation, suggesting a more complex mechanism of action than simple TNF inhibition. The peptides appear to act by modulating the inflammatory activity of macrophages, inhibiting secretion of cytokines including but not limited to TNF. Since these molecules work via a different mechanism of action from anti-TNF antibodies, they could work very well as a complement to TNF blockade therapy, helping to improve efficacy, when administered concurrently, beyond that already seen.

## 5. Materials and Methods

### 5.1. Reagents

Micelles: amphiphiles were synthesised as described in reference [1]. Laurel sarcosine, octadecyl rhodamine, hexadecyl fluorescein, octyl glucoside and phosphate buffer tablets were purchased from Sigma UK Ltd. (Poole, UK). Phosphatidyl choline was obtained from Lucas Meyer (Chester, UK). Cholesterol oleate (tritium labelled) was purchased from Amersham International Ltd. (GE Healthcare, Bucks, UK), and oleyl sarcosine was synthesised by Peptide & Protein Research Ltd., UK (Fareham, UK).

Peptides: cyclic peptides were synthesised by Peptide & Protein Research Ltd., UK (Fareham, UK) using standard methods (solid phase Fmoc chemistry, followed by cyclisation in solution). This company also synthesised the racemic and chiral lipoamino acids employed. Transcutol (diethylene diglycol), hexafluoroisopropanol (HFIP) and β-hydroxypropyl cyclodextrin (CD) were all purchased from Sigma UK Ltd.

J774A.1 Cell culture: Dulbecco’s Minimal Essential Medium (DMEM) and foetal bovine serum (FBS) were purchased from Invitrogen (Paisley, PA4 9RF, UK). Pen/Strep solution (25 U/mL) and l-glutamine (20 mM) were obtained from Gibco UK (Paisley, PA4 9RF, UK). Cholera Toxin B fragment (CTB) and *E. coli* lipopolysaccharide (LPS) were purchased from Sigma UK Ltd. The ELISA kit for detection of Tumour Necrosis Factor (TNF) was purchased from R&D Systems Ltd. (Abingdon, UK). Cells of the J774A.1 line were purchased from the European Cell Culture Collection (PHE, Porton Down, Salisbury SP4 0JG, UK) at passage number 14.

For the in vivo studies, the antibody cocktail and lipopolysaccharide were purchased from Chondrex Inc. (Redmond, WA98052, USA, catalogue number 53100). In the first study, the cocktail comprised a combination of four anti-collagen type II antibodies A2-10, D1-2 G. D8-6 and F10-21. In the second study, and additional antibody was added to the cocktail, D2-112).

### 5.2. Preparation of Micelles

Constructs were each dissolved in a mixture of dichloromethane/methanol 2:1 (*v*:*v*) at a concentration of 10 mg/mL, with warming at 37 °C. A co-amphiphile solution of lauryl or oleyl sarcosine was prepared in the same solvent at a concentration of 20 mg/mL. Equal aliquots of each of the amino acid constructs required for a given micelle were mixed together in a glass vial, to which an equal volume of lauryl sarcosine solution was added. After mixing on a roller mixer at room temperature, the solvent was removed either by allowing to evaporate slowly in a well-ventilated atmosphere, or under a stream of nitrogen. The dry residue was exposed to vacuum (1 mbar) overnight, then dispersed in phosphate-buffered saline, with vortexing. The total concentration of amino acid constructs was adjusted to be 1 mg/mL for testing in cells, 0.4 mg/mL for testing by scintillation proximity, or 0.2 mg/mL for FRET measurements. In some cell culture experiments octyl glucoside or soya phosphatidyl choline were used as alternative co-amphiphiles, under the same conditions as described above.

The amino acids selected to form the pool of ten are intended to be representative of all the major classes of residue involved directly in binding interactions, i.e., hydroxylic, lipophilic, positive, negative, amide bearing and aromatic. The one letter codes for the ten amino acids chosen are E—glutamate, F—phenylalanine, H—histidine, K—lysine, Q—glutamine, R—arginine, S—serine, W—tryptophan & Y—tyrosine. Prior to use, micelles were extruded through a 0.2 micron straight-pore Anotop 10 inorganic membrane filter (Whatman Gmbh, Dassel, Germany) to control the upper size limit.

### 5.3. Cell Culture (J774A.1 Macrophage Cell Line)

Cells between 40 and 60 passage number were maintained in culture at 37 °C in DMEM supplemented with 10% Foetal Bovine Serum (FBS), 1% Pen/Strep solution and 1% glutamine in a 5% CO_2_/air atmosphere. For each experiment, cells were scraped gently from the surface of the culture vessel and resuspended in medium without FBS, at a concentration of 0.2 × 10^6^ cells/mL. The cell suspension was then transferred to each well of 24-well cluster plates (1 mL per well). The cells were incubated overnight to give a sub-confluent, adherent monolayer, whereupon the medium was replaced with fresh medium containing micelles at the desired concentrations. The cells were incubated overnight, and the TNF concentration in the supernatant was measured the following day using a sandwich ELISA kit. In some experiments, immunostimulants (CTB, 10 micrograms/mL or LPS, either 30 or 60 nanograms/mL final concentration) were added to the wells prior to overnight incubation, four hours after administration of the micelles. All determinations were conducted in triplicate wells, and reported as the mean and standard deviation.

### 5.4. LPS-Stimulated Rat Model

Adult female Sprague-Dawley rats (6 per group) were weighed and then given a single intraperitoneal injection of vehicle (phosphate buffered saline) or test material at 2 mg/kg. Thirty minutes later, animals received a second intraperitoneal injection of either saline or 1 mg/kg LPS. Blood (~500 μL) was harvested into syringes containing 3.8% sodium citrate, from the tail vein of all animals 45 min, 1.5 h, 2.5 h and 24 h after the intraperitoneal saline or LPS injection. Cells and plasma were separated by centrifugation. After 24 h, all animals were weighed and euthanised. A peritoneal lavage was performed immediately with 2 mL of sterile saline. Heart, liver, spleen, kidney and lung tissue were collected into fixatives. This study (protocol number 0408-045A) was conducted with the approval of the Sydney & Royal North Shore Hospital Joint Animal Care & Ethics Committee.

### 5.5. CAIA Model in Mice

The time-course for the Collagen Antibody-Induced Arthritis (CAIA) model in mice is shown in Figure 8 of the results section. Female DBA mice were employed (19–20 g each at the start of the experiment, ten animals per group). The initial stimulus, comprising a cocktail of anti-collagen antibodies (1.5 mg, provided by Chondrex Inc.—see Reagents section 2.1) was administered i.p. on day 1, followed by a non-specific inflammatory stimulus (25 ug *E. coli* 0111:B4 lipopolysaccharide from Chondrex Inc.) two days later, whereupon inflammation in all four paws developed rapidly, and was maintained for up to ten days before termination. In the first study, treatments were administered as a single i.p. dose on day 1 (Humira^®^), or as a repeat i.p. administration from day 1–10 (lexicon peptide) as shown in Table 1 in the Results section. Volumes injected were 0.1 mL in all cases. In the second study, an identical protocol was followed, except that the first lexicon dose was administered on day 1. Blood samples were taken via tail vein prick at three time points on day 3, and samples and organs were taken for histology after sacrificing the animals at the end of the experiment. Plasma was prepared using heparin (5 U/mL) and centrifugation at 6000× *g* for 5 min. Organs were frozen immediately in liquid nitrogen or placed in phosphate buffered saline paraformaldehyde 4% (PFA) solution for subsequent histology. On a daily basis, animals were weighed, footpad thicknesses measured with a micrometer screw gauge without paw compression, and a clinical score assigned to reflect the general severity of the condition; for each of the four paws, a score of one was assigned for each of the joints affected (metacarpal/metatarsal/digit) on visual appearance, and the scores summed, resulting in a total potential score of 12 for animals most severely affected. These studies were conducted with the approval of the Ethics Committee of the Regierungs Präsidium Tübingen in compliance with German Federal Regulations under file SYN_02_14.

## Figures and Tables

**Table 1:** Doses administered in the first CAIA study.

Agent		Dose (mg/kg)	Administration
1	None	-	
2	Lex 5.2	0.8	Days 0–10
3	Lex 5.2	2.0	Days 0–10
4	Lex 5.2	5	Days 0–10
5	Humira (anti TNF ab)	10	Day 1
6	Naive	-	
7	Lex 5.2 *	0.32	Days 0–10
8	Lex 5.2 *	15	Days 0–10
9	None *	-	
10	Naïve *	-	

* Experiment conducted 2 weeks after the first set, in the same cohort of animals.

**Table 2:** Doses administered in the second CAIA study.

Agent		Dose (mg/kg)	Administration
1	Lex 5.2	-	Days 0–10
2	Lex 5.2	0.025	Days 0–10
3	Lex 5.2	0.05	Days 0–10
4	Lex 5.2	0.1	Days 0–10
5	Lex 5.2	0.2	Days 0–10
6	Lex 5.2	0.3	Days 0–10
7	Lex 5.2	0.6	Days 0–10
8	Lex 2.6	0.3	Days 0–10
9	Humira (anti TNF ab)	10	Day 1
10	Enbrel (anti TNF)	10	Day 1
